# Design and Validation of PACTUS 2.0: Usability for Neurological Patients, Seniors and Caregivers

**DOI:** 10.3390/s25196158

**Published:** 2025-10-04

**Authors:** Juan J. Sánchez-Gil, Aurora Sáez, Juan José Ochoa-Sepúlveda, Rafael López-Luque, David Cáceres-Gómez, Eduardo Cañete-Carmona

**Affiliations:** 1 Departamento de Ingeniería Electrónica y de Computadores, Universidad de Córdoba, Edificio Leonardo Da Vinci, Campus de Rabanales, 14071 Córdoba, Spain; aurora.saez@uco.es (A.S.); ecanete@uco.es (E.C.-C.); 2 Instituto de Neurociencias, Hospital Cruz Roja, P.º de la Victoria, 14004 Córdoba, Spain; 3 Departamento de Ciencia de la Computación e Inteligencia Artificial, Universidad de Córdoba, Edificio Marie Curie, Campus de Rabanales, 14071 Córdoba, Spain; dcaceres@uco.es

**Keywords:** stroke, gamification, gamified devices, rehabilitation, gamified therapy, neurorrehabilitation

## Abstract

Stroke is one of the leading causes of disability worldwide. Its sequelae require early, intensive, and repetitive rehabilitation, but is often ineffective due to a lack of patient motivation. Gamification has been incorporated in recent years as a response to this issue. The aim of incorporating games is to motivate patients to perform therapeutic exercises. This study presents PACTUS, a new version of a gamified device for stroke neurorehabilitation. Using a series of colored cards, a touchscreen station, and a sensorized handle with an RGB sensor, patients can interact with three games specifically programmed to work on different areas of neurorehabilitation. In addition to presenting the technical design (including energy consumption and sensor signal processing), the results of an observational study conducted with neurological patients, healthy older adults, and caregivers (who also completed the System Usability Scale) are also presented. This usability, safety, and satisfaction study provided an assessment of the device for future iterations. The inclusion of the experiences of the three groups (patients, caregivers, and older adults) provided a more comprehensive and integrated view of the device, enriching our understanding of its strengths and limitations. Although the results were preliminarily positive, areas for improvement were identified.

## 1. Introduction

Stroke is a global problem [1] that requires early rehabilitation to reduce the degree of patient disability [2,3]. The sequelae that patients with stroke may experience vary and cannot always be predicted. The most common symptoms include cognitive and physical deficits, depression, tremors, dysphagia, and hemiplegia of the limb [1,4,5]. These conditions significantly alter the quality of life of stroke survivors, necessitating rehabilitation therapy. For rehabilitation to be effective, it must be prolonged and intensive [6,7]. However, many patients experience high levels of demotivation [8].

The literature shows that gamification is a solution to counter both intensive repetition and low motivation [6,9,10], which often emerges as a complement or alternative to conventional therapy [9,11].

The implementation of gamification (the incorporation of game elements into non-gaming contexts [9,12]) in stroke rehabilitation systems has been reported in various ways in the literature, generally yielding positive results [13]. Serious Games (SG) are games whose primary objective is different from entertainment and are more oriented toward achieving concrete goals such as education or health promotion [11,14]. However, designing these gamified systems typically presents a challenge as it requires a multidisciplinary approach [1,8].

This study presents the design and development of PACTUS (2.0), a gamified system for neurorehabilitation that is mainly focused on stroke patients. It highlights all phases undertaken, necessary design justifications, and device validation involving patients, healthy older adults (more susceptible to stroke [15]), and caregivers.

The remainder of this study is organized as follows. Section 2 reviews works related to the device presented here, emphasizing the innovations of this proposal, which builds on a previous prototype described in [16]. Section 3 details the design criteria, game dynamics, and description of each game. Section 4 describes the design and implementation of this new version of PACTUS, presenting the hardware resources, a study of the new sensor used, and device software. Section 5 presents the experimental design used to validate this version of the device (PACTUS 2.0). Section 6 presents the results of the study. Finally, Section 7 discusses these results, and Section 8 offers the conclusions of the study and outlines future directions.

## 2. Related Work

This section presents the background of PACTUS 2.0 found in the literature. Next, previous versions of PACTUS are discussed, pointing out their limitations. Finally, the new version’s proposal is detailed, explaining how it improves upon its predecessors.

### 2.1. Antecedents

Numerous gamified solutions are aimed at assisting the stroke rehabilitation process. The most relevant studies are reviewed below.

First, in [17,18,19,20] they use Cellulo commercial tangible robots for gamified stroke rehabilitation. These robots were designed to represent interactive objects that reside in a [21] plane. In [17], the authors concluded that the use of this game influences the amount and type of upper-extremity movement of the player, which is key to rehabilitation. In [18], the results supported several design approaches, demonstrating that the game setup facilitated the performance of repetitive exercises in a motivating manner. On the other hand, in [19], the authors emphasized that this solution benefited more patients with a mild disability, while in [20], they oriented the objectives more in terms of compensations in the patients’ movements.

The EDNA-22 or “Elements” system is presented in [22,23]. This gamified device was designed to aid cognitive and motor recovery following stroke. It consists of tangible elements and an interactive screen and provides feedback that encourages patient engagement. Both studies noted enhancements in the physical and cognitive abilities of the patients.

In [24], the authors introduced Ergotact, a device featuring an interactive display, a tangible component, and a Serious Games (SG), which showed improvements in upper-limb function, although this was not statistically significant. The authors of [25] created six virtual cell-phone games designed to enhance visuospatial memory, showing significant promise for evaluating cognitive impairment and tracking changes in patients with stroke. In [26], inertial sensors were utilized to record the upper-limb movements of stroke patients, revealing considerable potential in the gamified system they developed. The study in [27] introduces a table equipped with a large virtual screen and tangible elements for engaging with simple games in both competitive and collaborative multiplayer modes, which fostered social and cognitive improvements. In [28], a preliminary study was conducted on a robotic table with Virtual Reality (VR) games, yielding encouraging results for enhancing upper-extremity function. Finally, Ref. [29] describes the use of a computer screen and electromyography sensors for grip rehabilitation, also showing promising outcomes.

### 2.2. Previous PACTUS Systems

The background literature preceding PACTUS 2.0 has been reviewed. This new version was the result of two previous design iterations. PACTUS 0.0 was the initial concept framework that led to PACTUS 1.0, as detailed in [16].

PACTUS 0.0 features a handle containing a digital infrared sensor that outputs a logical 0 or 1 depending on whether it detects white or black on a card, respectively. A single game board with a closed loop for upper-limb movement was used, and a simple 2D avatar on an LCD provided feedback. After reviewing this setup with an engineering team, it became clear that there was room for a more comprehensive solution.

PACTUS 1.0, which uses several A3 laminated cards, each containing up to three game boards. The patients performed repetitive exercises with audiovisual feedback and a point system. With a simple interface, multiple game modes, and adjustable difficulty, it proved usable and safe for chronic stroke patients (those more than six months post-stroke [30]). The interaction was achieved via a sensorized handle using the analog TCRT5000 infrared sensor (Vishay Semiconductors, Malvern, PA, USA), which allowed us to implement various shades of gray in a series of colored circuits printed on game boards. Feedback from the clinical team then guided improvements incorporated into the new PACTUS prototype.

However, PACTUS 1.0, had several limitations during the validation process. First, the diameter of the handle was too narrow. This made it difficult for people with grip problems, such as healthy elderly individuals and stroke patients, to interact with the system. This was a significant issue because a poor grip on the handle resulted in inaccurate readings from the infrared sensor. This caused the audible alarm system to sound continuously, which was annoying. Additionally, maintaining a poor grip was uncomfortable and prevented the correct execution of therapeutic movements to improve mobility of the affected limbs. However, the programmed cognitive game (Simon Says) was too complex for patients in the early stages of rehabilitation and could not be used in this version of PACTUS.

Therefore, advancing the iterative process of PACTUS is necessary to find a versatile, low-cost solution that would be the most effective gamified tool for post-stroke neurorehabilitation.

### 2.3. Proposal

The new version of PACTUS has improved the field of gamified neurorehabilitation for stroke. First, it stands out for its variety and versatility, since it has three different SG, which avoids habituation to a single game and facilitates the execution of multiple movements in the upper extremities [10]. This represents an advantage over previous versions reviewed in the literature [17,18,19,20,22,23,24], while also providing a more user-friendly interface.

Although it will be explained in more detail later, PACTUS 2.0 consists of a set of colored cards that can be arranged in different configurations. These cards replicate the therapeutic movements that a patient must perform. The user can interact with the SG by using a sensorized handle to recognize each color. This flexibility offered by PACTUS 2.0, when configuring the game board, represents a step forward compared to other gamified systems reviewed in the literature (Section 2.1). Not only because of the flexibility, but also because of the customization it provides therapists, allowing them to adapt the game board according to the specific movements each patient actually needs. By combining colors in different configurations, the system makes it possible to program transversal, longitudinal, opening, or closed-loop movements (among those considered the most appropriate) tailored to the physician’s recommendations. Thus, without changing the essence of the game (thus eliminating the added difficulty of requiring the patient to learn new internal game rules), the patient can interact with the same game in different ways.

This is a significant improvement over previous devices (such as Cellulo [17,18,19,20], among others [24,26]), which have more preset movements. PACTUS 2.0’s flexibility directly addresses one of the main limitations identified in the literature, as just seen: most gamified rehabilitation systems only offer fixed or minimally adjustable tasks.

In addition, PACTUS is characterized by its low cost and uses simple components that provide a cost-effective solution compared to other motorized or commercial systems [22,23,28]. The need to design accessible low-cost solutions has been previously highlighted in the literature [31,32,33], with such devices serving as valuable complements to conventional therapy [34]. Although many low-cost solutions have been implemented through commercial video games [10,35] (such as those using the Wii console (Nintendo) [36,37,38] or Kinect (Microsoft) [39]), they are primarily oriented toward leisure and entertainment rather than the execution of therapeutic movements [40,41]. This limitation is overcome in PACTUS 2.0, in two ways: first, by offering a design with low-cost and simple hardware and components (a consideration emphasized in [41]), and second, by enabling full customization of the movements that the patient must perform, as previously highlighted.

Compared to earlier versions, PACTUS 2.0 offers significant improvements in the robustness of color readings, an upgraded patient handle, and an enhanced Graphical User Interface (GUI). In addition to the color read by the sensorized handle on the cards, a rotary knob was incorporated to control one game. This allows for not only movement of the upper limb, as in the previous PACTUS, but also incorporates a new tangible element, the rotary knob, for the rehabilitation of fine psychomotor skills of the hand. This is because the movement of the avatar is achieved by moving the rotary knob wheel with two fingers. This is also novel compared to previous studies because it incorporates different inputs in the system that allow for the physical rehabilitation of multiple body parts.

**Figure 1:**
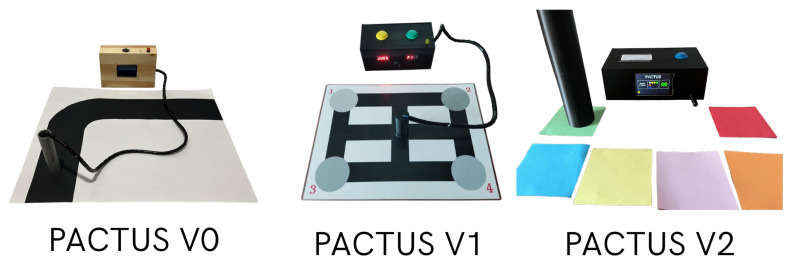
Different iterations of PACTUS: version 0 (V0), version 1 (V1), version 2 (current, V2).

Therefore, PACTUS 2.0, replaces the infrared sensor with an RGB sensor. The grip was redesigned for greater stability and robustness and wirelessly eliminated cumbersome cables for the patient. Now, the handle reads a wider range of colors and sends the color identifier (color ID) wirelessly to the central station, which processes it and drives three Serious Games (SG) designed for neurorehabilitation based on the detected color. A sample of the three devices is shown in Figure 1.

This new version of PACTUS 2.0 aims to overcome the limitations of its predecessors not only at a technical level but also at a therapeutic level. With new games and inputs to interact with them, the goal is to ensure that the patient does not get bored with the system. The same inputs are intended to assist in achieving therapeutic goals, expanding the possibilities of movement with hemiplegic limbs. In addition, the incorporation of a better GUI aims to help enhance arm–eye coordination through avatars in SG, as well as to make the interface more intuitive and user-friendly.

This iterative process was conducted in collaboration with a multidisciplinary team of neurorehabilitation physicians, occupational therapists, and engineers specializing in electronics and computer science. As noted previously [1,8], this collaboration is one of the key factors in designing new neurorehabilitation therapies.

## 3. PACTUS 2.0 System

**Figure 2:**
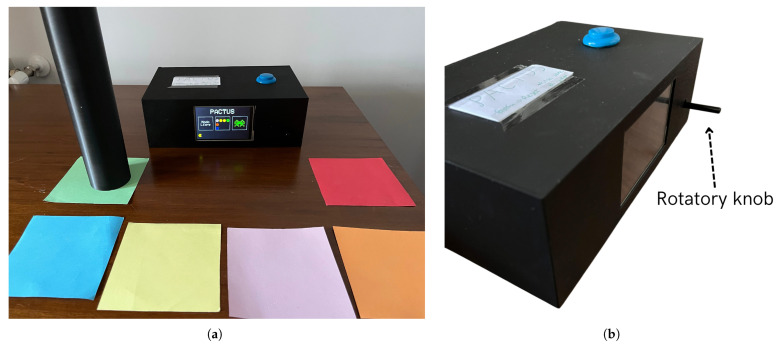
PACTUS 2.0 system overview. (**a**) PACTUS 2.0 and the main menu (in which one of the three programmed games can be selected); (**b**) PACTUS 2.0 additional input (a rotary knob for fine motor skills rehabilitation with the fingers of the hand).

In this section, an introduction to the new PACTUS prototype is presented, discussing both design considerations and the games themselves. An image of the assembled and powered-on devices is shown in Figure 2.

As can be seen in the figure, the system features a touchscreen that allows the user (patient or physician) to select the desired game mode). Then, on the same screen, visual feedback for each of the presented games was displayed.

The main objective of PACTUS, as noted in [16], is to enable the patient to perform therapeutic movements in a safe, intensive, and repetitive manner. The incorporation of game elements also aims to foster adherence and motivation among patients. In addition to the sensorized handle, the system also includes (as previously mentioned in Section 2.3) a rotary knob that allows the user to interact with their fingers in some of the programmed games (to be presented later). An image of the new input is shown in Figure 2b.

**Figure 3:**
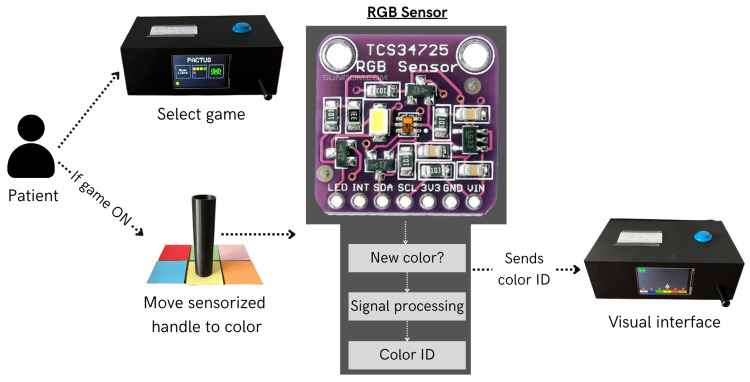
PACTUS 2.0 architecture, showing the main processes during patient–device interaction

Figure 3 illustrates the architecture of the system.

### 3.1. Design Considerations

The literature presents various design criteria for gamified systems. The main considerations taken into account are summarized in Table 1.

To achieve the objectives described above, a number of key design considerations have been considered for this new, improved version of PACTUS. Building on enhancements proposed by the medical team at the Instituto de Neurociencias de la Cruz Roja de Córdoba, mentioned in [16], focused on an economical gamified rehabilitation system. Additionally, the grip has been improved by increasing the diameter and adding a small base to prevent the patient from tipping it over owing to an improper hold. Another outstanding issue was the basic feedback provided by the PACTUS 1.0. This was enhanced by incorporating a touchscreen instead of a TM1637 display. The considerations in the mechanical and hardware aspects of the system are intended to improve its usability and appeal.

### 3.2. Device Dynamics

This new version of PACTUS offers several new dynamics compared to the previous version, which will now be discussed. The first is the replacement of the infrared sensor with an RGB sensor, which has provided the opportunity to change the sheets from unchangeable movement sets to the possibility of customizing the patient’s movements for each set. Individual colored geometric shapes are now used, which allow for interaction between the patient and game when the sensor reads the color.

This also adds an extra level of difficulty to the games if the therapist deems it necessary, since the arrangement of the colors can differ according to the patient’s needs, as can the size of the color cards (the smaller they are, the more accurately the patient must match the correct color).

**Figure 4:**
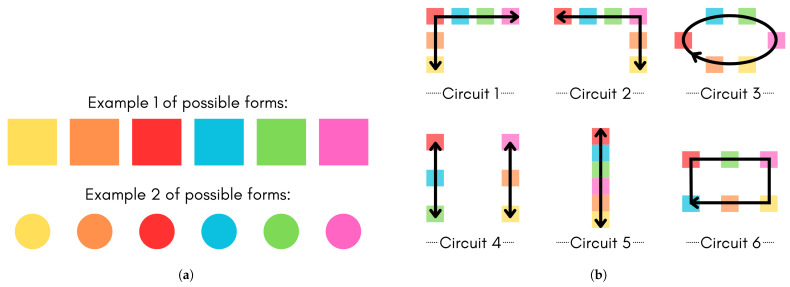
Comparative view of geometric shapes and circuit configurations. (**a**) Different possible colored geometric shapes; (**b**) possible configurable circuits for upper-limb movements (arrows).

An example of the different geometric shapes that can be used is shown in Figure 4a, whereas some of the possible movements that can be programmed are shown in Figure 4b. This demonstrates the versatility of PACTUS in customizing the therapeutic movements for each patient. It also provides an advantage over systems found in the literature, which usually have closed circuits and are not customizable.

### 3.3. Proposed Games

Three distinct games were implemented in this version of PACTUS (https://youtu.be/qqMmGMtitKE, accessed on 30 June 2025). Among the elements considered in the SG design found in the literature, the following were selected: difficulty (based on color size, as mentioned above) [44,55,56], goals and challenges (which define what the patient must do in each game to properly carry out the session) [55,57,58,59], feedback (primarily visual) [57,59,60,61], speed (regarding the execution speed of movements to interact with one of the games) [55,62], lives [55,63], points [55,58,59], simplicity [44,60,64], Non-Playable Characters (NPC) [65], and avatars [57,59,63]. The implemented games and their characteristics are shown in Table A1 (Appendix A).

## 4. Design and Implementation

This section details the design and implementation of PACTUS 2.0, including the hardware resources and methodology used to select the most suitable RGB sensor for the device, as well as the firmware that integrates PACTUS 2.0.

### 4.1. Hardware Resources

**Figure 5:**
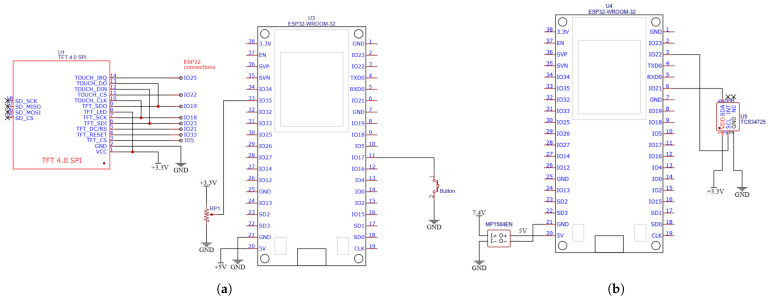
Electrical diagrams of PACTUS 2.0 components (main station and sensorized handle). (**a**) Electric schematic of the station; (**b**) electric schematic of the sensorized handle.

This section presents the hardware elements used in both the station and sensorized handle. Figure 5 shows the schematic of both. The hardware components used were as follows.

- ESP32-WROOM-32 module (Espressif Systems, Shanghai, China): A low-cost, low-power microcontroller with integrated Wi-Fi and Bluetooth, widely used in IoT and embedded applications.
- TCS34725 RGB color sensor module (Hongkong Yingli International Trading Co., Limited, Hong Kong, China): chosen for its high-precision RGB measurements with a built-in IR filter and 16-bit ADCs per channel. Its I^2^C interface simplifies the wiring and minimizes the pin count, enabling a compact handle design without compromising direct, linear, and stable readings.
- Touchscreen TFT display: A 2.8-inch ILI9341 TFT LCD display module (Shenzhen HiLetgo E-Commerce Co., Ltd., Shenzhen, China) was initially chosen and then replaced by 4-inch TFT LCD touch display module, 480 × 320, ST7796S (Shenzhen Hongshuyuan Technology Co., Ltd. [LAFVIN], Shenzhen, China). It communicates with the microcontroller via the SPI.
- Arcade button (sourced from Electrónica Caballero, Córdoba, Spain): Used to return to the main menu during a game session without power-cycling the system.
- Potentiometer 4.7 kΩ (sourced from Electrónica Caballero, Córdoba, Spain): Used to enhance the rehabilitation of the fine psychomotor skills of the fingers by controlling one of the games through this element.
- Battery (sourced from Electrónica Caballero, Córdoba, Spain): Two 18650 lithium-ion batteries, each with a capacity of 2500 mAh and voltage of 3.7 V, were used to power the sensorized handle. The display station will either use a 20,000 mAh power bank (external battery) or be directly connected to the mains using a 220-5 V transformer (UGREEN 17W USB wall charger; Ugreen Group Limited, Shenzhen, China) and a micro USB cable for the ESP32.
- MP1584EN (Hongkong Yingli International Trading Co. Limited [DollaTek], Hong Kong, China): This synchronous step-down integrated circuit converts input voltages ranging from 4.5 V to 28 V into an adjustable output starting at 0.8 V and delivering up to 3 A. Owing to its high-frequency (1.5 MHz) and internal MOSFETs, it provides a compact solution with overcurrent and temperature protection. It regulates the 7.4 V from the batteries to 5 V, which supplies ESP32 in the sensorized handle.

### 4.2. Station Firmware

The TFT_SPI library (2.5.43 version) was used for display handling. The design of this component was modified from the initial proposal. Initially, a 2.8-inch (240 × 320) TFT display was tested, but discarded because the sprites appeared too small. This could potentially pose a problem for older adults and those with impaired vision. This display used ILI9341_DRIVER from the library. It was then replaced with a current 4-inch (480 × 320) TFT display, which uses ST7796_DRIVER. Some dimensions were readjusted (as expected) to adapt to the original program from a 2.8-inch to a 4-inch screen. Moreover, for wireless data transmission between the sensorized handle and TFT display station, the ESP-NOW protocol is employed. This protocol has been used previously in the literature and has been proven to be secure and low-power [66,67].

#### 4.2.1. Graphical User Interface (GUI)

**Figure 6:**
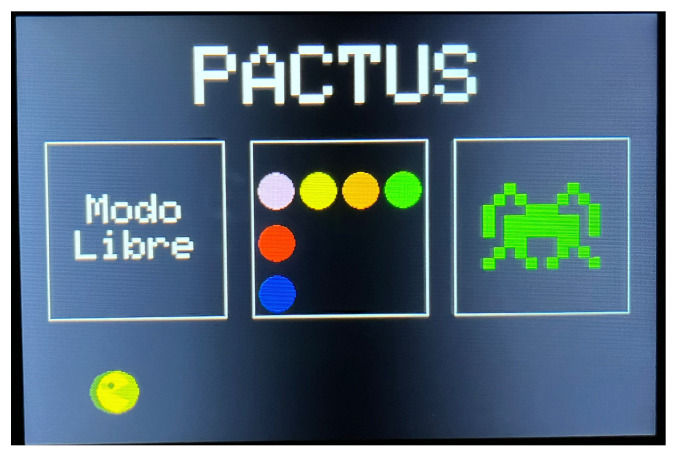
PACTUS 2.0 main menu, with the option to select (by tapping the screen) any of the games. It features a small PACMAN animation to make the GUI more user-friendly.

The GUI features a simple interface in which the user can choose among three games from the main menu by tapping on each game’s grid or sprite. To make it more attractive, a PACMAN avatar continuously traversed the bottom of the screen. See Figure 6.

The prototype included a physical push button wired to an external interrupt on the ESP32 to provide an immediate “escape” back to the main menu without power-cycling. This is essential in the free mode, where the game logic lacks a natural end condition, and allows the user to abort any session at will.

#### 4.2.2. State Machine

The high-level logic is structured as a three-state finite-state machine (S0–S2). State S0 serves as the root: it initializes after boot and exiting any game, renders the main menu, and waits for a touch that selects a game. An explicit transition to S1 occurs as soon as the user taps one of three active menu areas. S1 is a brief pre-start state; for Game 2 (Feeding PACMAN), it displays setup instructions, while for all games, it shows a 3 s countdown with a color change (red → yellow → green) to give the player time to prepare. When the countdown ended, the system entered S2, where the main loop of each game ran.

Two asynchronous events can transition the system from S2 back to S0: the user’s push-button request (captured by the interrupt described above), and each game’s local end-of-game condition.

**Figure 7:**
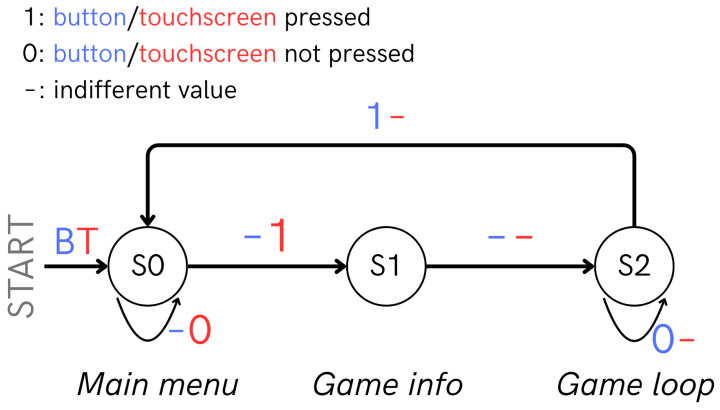
PACTUS 2.0 state machine (B is the blue button, T is the touchscreen).

This design (shown in Figure 7) simplifies resource management, prevents inter-game blocking, and allows each graphics or communication module to initialize cleanly between states, yielding deterministic, extensible firmware behavior.

### 4.3. Power Supply Subsystem

PACTUS 2.0 uses two independently powered subsystems: (1) the station with the TFT touchscreen and (2) the sensorized handle powered by two 18,650 cells.

- For the station, as mentioned above, a 20,000 mAh (77 Wh) external power bank providing 5 V and 2.4 A was used. However, it can also be used with a 220 V to 5 V adapter and micro-USB cable, allowing for direct connection to the main power when desired.
- The sensorized handle uses two 3.7 V 18,650 batteries in series (theoretical 7.4 V), regulated by an MP1584EN module to supply the ESP32 VIN pin. The ESP32 board then uses its onboard AMS1117 linear regulator to step 5 V down to the 3.3 V required [68], also protecting against voltage fluctuations.

The study of battery consumption by the handle is crucial for establishing the lifespan of the system before exhausting the charging cycle. For this purpose, Section 5.1 will describe the design of how the study of the handle’s energy consumption was conducted.

### 4.4. PACTUS Casing

**Figure 8:**
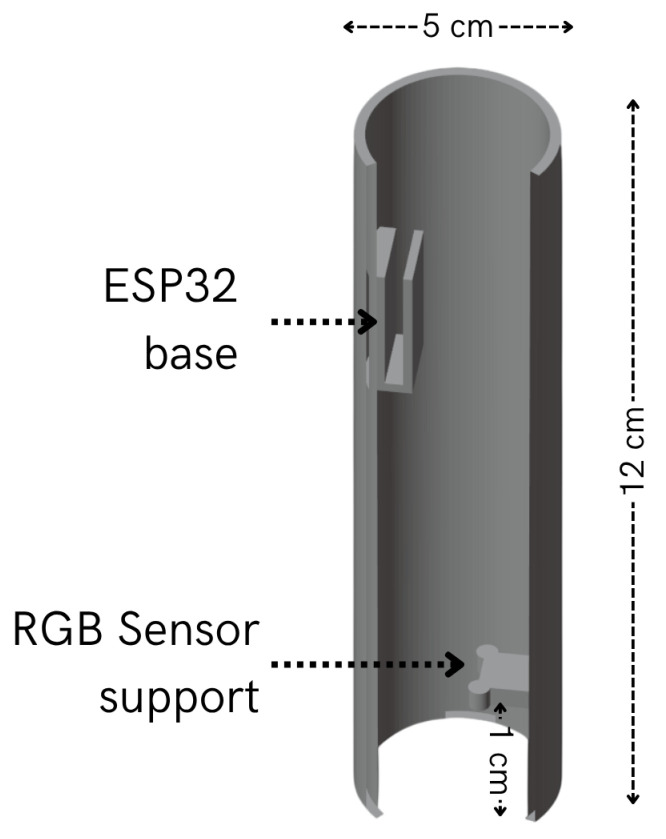
PACTUS 2.0 sensorized handle casing.

Both the TFT display station and sensorized handle had 3D-printed housings. The handle and its features are illustrated in Figure 8. As can be seen, the grip was 5 cm in diameter, the recommended size according to [24]. This size is intended to provide a more comfortable grip than PACTUS 1.0. The interior also offers a base to support the ESP32 and the sensor, which are separated from the surface by 1 cm.

**Figure 9:**
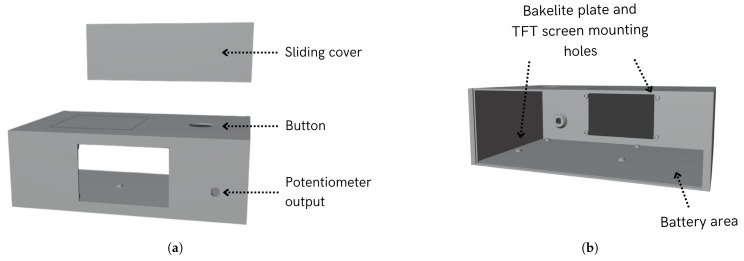
PACTUS 2.0 station casing. (**a**) Front view of the casing; (**b**) back view of the casing.

Figure 9 shows the front and back views of the central station. The system has anchorages for the Bakelite plate, where the electrical circuit is soldered, and for the TFT display. The system also had a sliding cover for accessing the interior, a hole for the button, and an exit output for the potentiometer.

### 4.5. RGB Sensor Processing

The TCS34725 sensor provides digital readings of the RGB components of colors. However, these signals must be processed for application to the PACTUS 2.0 system. This is mainly because transitions from one color to another may, in some instances, send the incorrect color ID. This would result in errors in executing and controlling the selected SG, thus worsening the gaming experience.

To simplify color reading and processing in the game, the raw sensor signal will be converted to a discrete level. Each color has been assigned an ID that the sensorized handle will send to the PACTUS station via ESP-NOW.

Later, Section 5.2 presents the design of the study carried out for the proper processing of the RGB sensor signal. The complete result of the signal processing can be found in Appendix B.

## 5. Experimental Design

This section presents in detail the experimental design used to validate PACTUS 2.0 as a whole. First, the study design for energy consumption is described. Next, the study of the RGB sensor signal processing is presented. Finally, the validation of the device’s usability is explained.

### 5.1. Design of the Study on Energy Consumption

To measure the consumption of the sensorized handle, a two-hour session of playing with PACTUS 2.0, was simulated in the laboratory. The battery voltage-level samples were taken every five minutes. This was intended to calculate the following values relevant for estimating battery discharge:

$Q_{\mathrm{op}}$—charge withdrawn.
$\Delta V_{\mathrm{op}}$—pack voltage.
$I_{\mathrm{avg}}$—average discharge current during the test.

These three values are relevant because they allow for the characterization of the real behavior of the battery during discharge. $Q_{\mathrm{op}}$ indicates how much effective charge was extracted and, therefore, what fraction of usable capacity was utilized. $\Delta V_{\mathrm{op}}$ reflects the voltage drop of the pack under operating conditions, which is directly related to the state of charge and perceived autonomy. $I_{\mathrm{avg}}$ summarizes the system’s average current demand, allowing for the estimation of both the discharge rate and typical operating conditions that define the battery life.

### 5.2. RGB Signal-Processing Design

**Figure 10:**
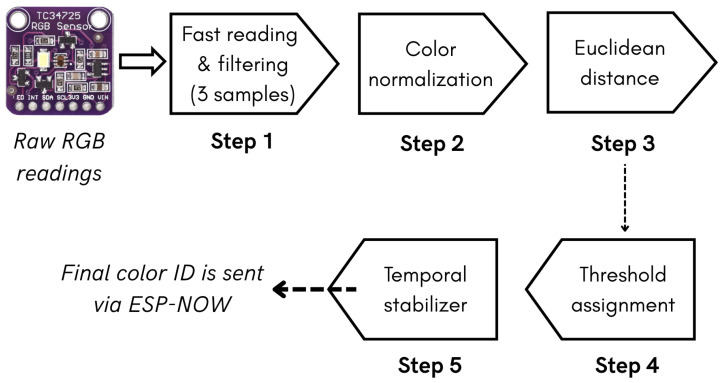
Diagram of the stages carried out during the processing of the RGB signal (full information: Appendix B).

The RGB signal processing was designed to ensure reliable, real-time color detection under different lighting and usage conditions. Several stages are carried out in which the raw signal is progressively processed to limit possible negative influences that could compromise the readings of the six established colors and, therefore, the gaming experience. Figure 10 illustrates how the RGB signal is processed at each stage. Full details of the signal processing can be found in Appendix B.

First, the sensor readings were refined by averaging three quick samples, which reduced the noise without adding perceptible latency. These values are then chromatically normalized by dividing each channel by the total intensity, so that classification depends only on hue rather than brightness level. Next, the generated normalized vector was compared with the six previously calibrated color centers (as described in Section 4.5) using a Euclidean distance metric. To avoid spurious classifications, the candidate color is accepted only if its distance is statistically below a specific threshold obtained during calibration. Subsequently, a temporal stabilizer requires the candidate to persist for 120 ms before being validated, which eliminates flickering caused by tremors or reflections. Finally, the stable color identifier (color ID) is transmitted via ESP-NOW every 200 ms, balancing the response speed with a low bandwidth and energy consumption.

This flow ensures robustness against noise, luminance variations, and possible false positives, while remaining stable and lightweight enough to run in real-time.

### 5.3. Usability Study Design with Subjects

This section presents the experimental design used to validate the usability of the PACTUS 2.0. The tests were conducted with caregiving staff at a nursing home and with several elderly participants (some with neurological disorders), a population that is more prone to stroke.

The testing protocol was as follows: (1) brief system introduction (≈5 min), (2) user testing by the participants (residents or caregivers) of the games and system dynamics (≈10 min, games: feeding PACMAN and Space Invaders), and (3) system evaluation (≈8 min). Thus, a single ≈20-min session was conducted per person. This preliminary evaluation methodology for a device consisting of a single session has been followed in other studies, such as [69,70,71,72]. Another objective of this single session was to avoid overtiring patients, who could experience greater fatigue during a prolonged session.

#### 5.3.1. Caregivers’ Subjects

For caregivers’ evaluation, the System Usability Scale (SUS) [73] was used. This is a brief standardized questionnaire that provides an overall usability score for any system. It consists of predefined statements to which users respond on a five-point Likert scale, simplifying both test administration and final score calculation. Through its questions, the SUS measures effectiveness, efficiency, and satisfaction in a concise format that minimizes participants’ time and cognitive load. In addition to this questionnaire, feedback was collected during and after the sessions.

Likewise, involving caregivers provides a complementary perspective on safety, ergonomics, and the relevance of game dynamics in care contexts, ensuring that the system is not only independently usable but also safely and effectively integrated into support and rehabilitation routines.

#### 5.3.2. Elderly Healthy and Neurological Subjects

The study with older subjects (some healthy and others with neurological conditions) provided necessary information to assess the current and preliminary usability of the device, with a view to improving it in future iterations. Initially, it was proposed that, in addition to the feedback received during and after the gaming session, participants would be given a four-question questionnaire to quantify how they viewed PACTUS 2.0, in terms of comfort, safety, usability, attractiveness, and motivation.

However, many older individuals (healthy or with neurological complications) had problems reading, understanding, and answering the questions. Therefore, it was decided that feedback from the session would be obtained only through the observational study conducted and brief individual questions to identify any complications encountered when using PACTUS 2.0, and areas for improvement.

Including older adults allowed us to identify age-related barriers (such as attention, memory, or coordination difficulties) and determine whether the gamified design and sensorized handle improved accessibility and motivation in this group. Table 2 presents the voluntary participants recruited for the usability study using PACTUS 2.0.

## 6. Results

This section presents the results of this study conducted in this work. First, the results from the experimental protocol (Section 5) were obtained with volunteer subjects to validate the usability of PACTUS 2.0 will be presented. Finally, the results of the experimental protocol were conducted with volunteers to validate the usability of PACTUS 2.0 will be presented.

### 6.1. Total Energy Consumption of TCS34725 RGB Sensor

This subsection presents the results for the energy consumption of the TCS34725 sensor.

**Figure 11:**
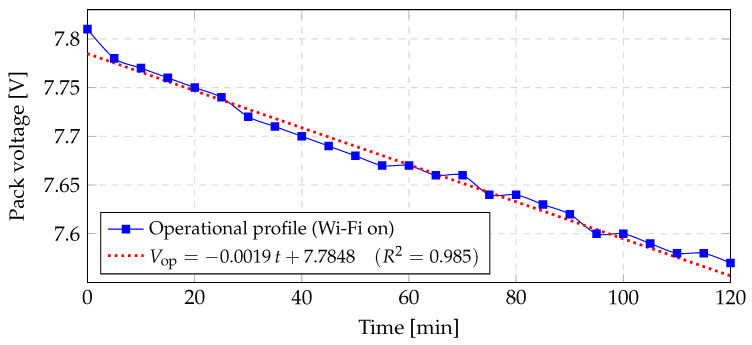
Discharge curve and linear fit for the *operational profile test.*

Figure 11 shows the discharge trace obtained while running the full firmware with Wi-Fi traffic. The dotted fit exhibits a slope of $-2.00\;\frac{\mathrm{mV}}{\min}$. This graphical result matches the analytically calculated $m_{\mathrm{op}}$ as follows:

The initial level remains V(0)=7.81 V; after 120 min, the pack settles at $V_{\mathrm{op}}\left(120\right)=7.57$ V. The numerical results are as follows:

$$\Delta V_{\mathrm{op}}=7.81-7.57=0.24\;V,$$

$$m_{\mathrm{op}}=\frac{0.24}{120\;\min}\approx 2.0\frac{\mathrm{mV}}{\min}.$$

With the same 7.4 V, 2500 mAh pack model, the charge drawn is:

$$Q_{\mathrm{op}}=C_{\mathrm{nom}}\;\frac{\Delta V_{\mathrm{op}}}{V_{\mathrm{nom}}}=2500\;\mathrm{mAh}\times \frac{0.24}{7.4}\approx 81\;\mathrm{mAh},$$

$$I_{\mathrm{avg}}=\frac{Q_{\mathrm{op}}}{\Delta t}=\frac{81\;\mathrm{mAh}}{2\;h}\approx 40\;\mathrm{mA}.$$

where:

$Q_{\mathrm{op}}$—charge withdrawn during the operational test (mAh);
$C_{\mathrm{nom}}$—nominal capacity of the two-cell pack (mAh);
$\Delta V_{\mathrm{op}}$—pack voltage drop observed in the test (V);
$V_{\mathrm{nom}}$—nominal pack voltage (two cells in series) (V);
Δt—duration of the test (h);
$I_{\mathrm{avg}}$—average discharge current over the Δt interval (mA).

After two hours of continuous operation, only about 3.2% of the pack’s usable capacity has been consumed, confirming that the system has ample reserve power for extended sessions or multiple rehabilitation cycles before requiring recharging.

### 6.2. PACTUS 2.0 Usability

This section presents results from the SUS and custom questions measuring usability, comfort, and motivation associated with PACTUS 2.0.

**Figure 12:**
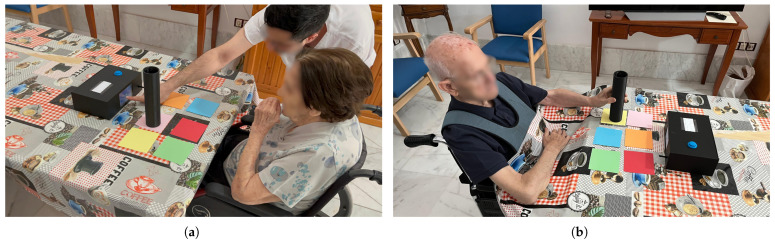
Volunteer subjects using PACTUS 2.0. (**a**) Volunteer 1 playing Feeding PACMAN with the help of caregiver 1; (**b**) volunteer 3 playing Feeding PACMAN.

The volunteer subjects who participated are shown in Table 2. In Figure 12, two of the volunteer subjects who took part in the study using PACTUS 2.0 can be seen. A total of three caregivers (48 years ± 12.53; mean ± SD) and eleven volunteer subjects (88.09 years ± 6.56), five of whom had neurological deficits, participated in the project. All provided written informed consent to participate in the observational study. Additionally, they authorized the use of the photos shown in this work.

**Figure 13:**
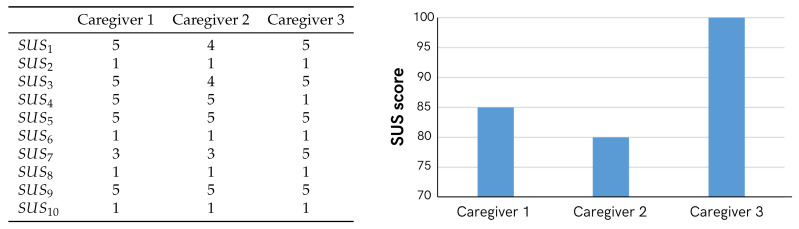
Individual SUS responses processed.

On the other hand, the SUS results can be seen in Figure 13. As for the values of the extra questions regarding motivation, satisfaction, appearance, and ergonomics, the 3 caregivers gave it the highest score.

## 7. Discussion

In this work, the design and development of PACTUS 2.0, a gamified rehabilitation device for neurological patients, are presented. Both the hardware and software validation of the device and its usability validation in a real-world setting have been demonstrated. The design steps proved optimal for the development of this new version of PACTUS.

Current gamified solutions present limitations in terms of the personalization of therapeutic movements [17,18,19,20]. This was achieved in this improved version of PACTUS by enabling the customization of movement circuits through the placement of colored cards with free-form patterns. Moreover, the observational study goes a step further by validating the prototype not only with patients, but also with caregivers. This approach has allowed both populations (caregivers and healthy older adults or those with neurological deficits) to provide highly valuable feedback for future device iterations.

### 7.1. What Does PACTUS 2.0 Offer?

In summary, the new features of PACTUS 2.0, compared to its predecessors and other literature, can be grouped into three categories.

1. Personalization and customization. As mentioned several times, PACTUS 2.0 offers the possibility to “program” different game boards depending on how the colored sheets are arranged. This allows the therapist to adapt the movement that each patient must perform according to their needs and the stage of the rehabilitation process. This flexibility, as previously noted in the literature, provides significant advantages compared with other systems with predefined movements [17,18,19,20,24,26].
2. Low cost and accessibility. In addition to complete customization of movements, PACTUS 2.0 ensures accessibility by using common, low-cost elements. This allows for possible implementation at both the clinic and home levels. Being a compact system, with small dimensions and easy implementation, PACTUS 2.0 has the potential to be integrated into home-based rehabilitation, following the guidelines established in [10,18]. Furthermore, the feedback received from caregivers is encouraging, as they agreed that the system is quite intuitive and requires no extensive prior training.
3. User engagement. Finally, PACTUS 2.0 has the potential to enhance patient motivation through variety and interaction. The platform integrates three different SG, prevents habituation to a single task, and encourages sustained engagement. The inclusion of a tangible rotary knob extends the rehabilitation focus from gross upper-limb movements to fine motor skills, while the touchscreen interface and visual feedback (e.g., PACMAN avatar) further increase immersion. The results of the usability study with caregivers and elderly participants, as discussed below, supported these design choices, showing high ratings for satisfaction and ergonomics.

In terms of usability, the device showed acceptable levels according to SUS. The lowest scoring items were related to independent use of the device. This was mainly because, during testing, neurological patients experienced various difficulties in identifying colors. For this reason, the caregivers considered that the device should be used primarily by a technician or qualified person while playing with PACTUS. This poses a disadvantage, as it requires the full attention of the care staff while the patient uses the device. It is also true that the advanced age and deficits of the volunteers may have influenced the results.

The feedback received has brought to light various limitations and future proposals, which will be discussed later. However, this preliminary usability study achieved its main objective: to establish a clear basis for building the next iteration of PACTUS. The caregivers were very optimistic about the device. They suggested additional issues with with SUS results. They observed that the device could not be operated autonomously by the patient. However, they have observed that a person accompanying the patient at home can easily operate the device. They were so optimistic that they requested a device to be implemented in the nursing home, as they also saw that patients and elderly people could benefit significantly from the games on a cognitive level. On the other hand, the stroke patient showed some difficulty in performing certain movements and quickly associating colors, but no incidents of any kind were reported, and he was able to perform the movements adequately. In general, it is true that some people experienced difficulties seeing game sprites, although this ultimately did not completely prevent them from playing. While the device initially used a 2.8-inch screen that was later replaced with a larger one (4-inch), this change has proven insufficient in preventing the potential vision-related issues previously mentioned in Section 4.2.

Regarding motivation, satisfaction, appearance, and ergonomics, all participants agreed to receive the highest score. This reflects the overall satisfaction of users with the device. Feedback from patients and healthy older adults was generally positive, although they experienced difficulties in identifying and recognizing the colors. In fact, the trials with the Space Invaders game had to be canceled because the patients’ response times to the new alien colors exceeded the game’s firing interval. Ultimately, the patients ended up playing the Feeding PACMAN game, which did not have the temporal response requirement.

### 7.2. Limitations of the Study

Although this study is preliminary and aimed to further improve the prototype toward a more comprehensive solution, the low number of caregivers, healthy older adults, and neurological patients may have influenced the results. Furthermore, only one patient with stroke participated in the study, which was the main pathology under investigation. This small sample size prevented generalization and statistical validation of the results, although it is true that the experimental study was not a clinical trial. Rather, the preliminary study aimed to identify possible design and methodological gaps to continue improving the system.

Despite these limitations, the results confirm the feasibility of PACTUS 2.0, as a gamified rehabilitation platform, and suggest that, with some adjustments (increasing the clinical sample size and calibrating game difficulty for tasks requiring rapid patient response), the system can be reliably and safely integrated into physiotherapy, occupational therapy, and cognitive therapy programs in both hospital and home settings. Thanks to this preliminary study, the feedback received, and the tests conducted, it was possible to identify the technical gaps in both the design and methodology. Thus, the next iteration of PACTUS is intended to overcome all these limitations, offering an even more effective, engaging, and safe tool for gamified stroke neurorehabilitation.

### 7.3. Future Work: PACTUS 3.0

Acknowledging these limitations, this subsection presents future research directions for the PACTUS system.

First, the next iteration of PACTUS (version 3.0) should include additional games with different genres and mechanics, allowing patients with diverse preferences to perform therapeutic movements with the SG aligned with their subjective and intrinsic interests.

PACTUS 3.0, should also continue to improve the sensorized handle to provide more and better inputs through which patients can interact. In addition, enhancement of the GUI could benefit patients with visual impairments. Additionally, PACTUS 3.0 will require an improved GUI that allows for clearer viewing of sprites and game elements to prevent issues with patient–game interaction, as reported in the observational study.

To validate the clinical significance of PACTUS 3.0, clinical studies should be conducted with a larger sample of neurological patients, mainly stroke survivors. Efforts should also be made to establish a control population to better quantify the clinical improvements induced by the system. In addition, more sessions will be held over several days using the device for more minutes per session.

Finally, in future studies, the potential inability of color-blind individuals to use the device should be considered a limitation. Thanks to this preliminary study, it has been possible to quantify this limitation, which future work aims to solve in the following ways: (a) offering games whose interaction is based not so much on [colored cardboard]—[game], but rather on the [shape of the cardboard]—[game], and (b) introducing other peripherals for user–system interaction. However, PACTUS 2.0 can be operated by people with this problem in the following ways: either with the game “Space Invaders” using the rotary knob, or by performing the movements of “Free mode” by moving the handle over the cards (although the feedback received may not be useful, the programmed repetitive movements would help in the rehabilitation process).

## 8. Conclusions

The work presented herein demonstrates that PACTUS 2.0 is a viable, low-cost, gamified platform for upper-limb and cognitive neurorehabilitation. Through comprehensive hardware and software redesign (including the adoption of the TCS34725 RGB sensor owing to its superior chromatic fidelity and signal-to-noise stability at clinically relevant distances), the system reliably identifies colored cues. It also drives three Serious Games (SG) tailored to fine motor and gross upper-limb exercises. Energy-profiling tests confirmed that even under continuous WiFi-enabled operation, the ESP32-based handle consumes ≈ 40 mA, drawing 3.2% of a 2500 mAh battery over two hours, thereby ensuring autonomy for extended therapy sessions without frequent recharging.

Usability evaluation with caregivers and older adults yielded acceptable System Usability Scale scores and high ratings for motivation, appearance, and ergonomics, although some users required assistance with rapid color recognition. These findings affirm that PACTUS 2.0 can be safely integrated into physiotherapy, occupational therapy, and cognitive therapy workflows, both in clinical and home environments.

Despite limitations (including the small cohort and the need to calibrate game difficulty for faster response tasks), the positive engagement, robust sensor performance, and low power collectively support further clinical trials with more stroke survivors. Future work will expand the sample size and explore adaptive game mechanics to maximize patient adherence and functional recovery.

## Figures and Tables

**Table 1:** Design considerations for PACTUS 2.0, highlighting the main design elements and how they have been implemented in the device.

Design Element	Refs.	Implementation
Comfort (ease of use of the system)	[35,42,43,44]	→ Improved sensorized tangible grip, increasing the diameter to 5 cm to allow for better grip.
Intuitive interface	[45,46,47,48,49,50]	→ The main touchscreen menu consists of three grids, each displaying a series of sprites (2D images used to represent game objects, characters, or elements). Tapping any grid launches the corresponding game. → A “Return to Menu” button has been added so that, if a session needs to be interrupted, the patient can go back to the main menu without power-cycling the entire system.
Robustness	[42,43]	→ Processing has been added to the RGB sensor readings, enabling robust output at all times. → The sensor has been shielded from ambient light to prevent interference with the readings [35].
Lightness and ergonomics of materials	[33,43]	→ The materials have been designed and developed on a 3D printer, ensuring that they are not only shock-resistant but also lightweight.
Accessibility (economic)	[32,35,42,46]	→ The aforementioned 3D printing and selection of hardware resources make PACTUS 2.0 economical.
Monitoring	[48,51,52,53]	→ The feedback provided allows the patient’s progress to be monitored during the playing session.
Non-invasive	[48,52,53,54]	→ By using a sensorized tangible grip, the patient only needs to hold it; there is no need to attach anything to their body or clothing. → All cables connecting the sensor to the station have been removed, unlike previous PACTUS versions, so nothing interferes with arm movements.

**Table 2:** Characteristics of caregivers and volunteer subjects

Caregivers			
Subject	Sex	Age	Formation
Caregiver 1	Male	35	Nursing Assistant (equivalent to Spanish TCAE)
Caregiver 2	Female	60	Geriatric Nursing Assistant
Caregiver 3	Female	49	Nursing Assistant (equivalent to Spanish TCAE)
**Volunteers’ subjects**			
Subject	Sex	Age	Neurological disorders?
Volunteer 1	Female	96	No
Volunteer 2	Male	72	No
Volunteer 3	Male	85	Moderate–severe Alzheimer’s disease
Volunteer 4	Female	93	Dementia
Volunteer 5	Male	89	Ischemic stroke (dysphagia, Brunnstrom Scale = 5, three months since affection), and Parkinson’s disease.
Volunteer 6	Female	90	Dementia, moderate Alzheimer`s disease, and Parkinson’s disease.
Volunteer 7	Male	92	No
Volunteer 8	Male	86	Meningitis (cognitive sequelae: partial memory loss)
Volunteer 9	Female	84	No
Volunteer 10	Male	94	No
Volunteer 11	Female	88	No

## Data Availability

Data supporting the reported results, including itemized System Usability Scale (SUS) scores, are contained within the article.

## Appendix A. Description of PACTUS 2.0 Games

**Table A1:** PACTUS 2.0 proposed games.

Game	Description & Objectives	Rehabilitation Domains	Image
Free mode	A PACMAN avatar simulates eating and changes color according to the detected color ID, offering immediate visual feedback. Patients can freely explore the recommended range of motion, repeat movements at their own pace, and gain motor confidence through positive reinforcement.	Motor: repetitive upper limb movements (free exploration of movements). Cognitive: very low cognitive load; mainly basic interaction and hand–eye coordination.	
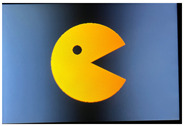
Feeding PACMAN	A hungry PACMAN must eat randomly colored fruits without repeats, moving from a static blue start to a green end. This trains hand–eye coordination, motor planning, and working memory as patients identify colors, direct the avatar, and remember pending fruits.	Motor: wide and repetitive upper limb movements to select colors (fruits); accuracy in positioning. Cognitive: working memory (avoid repeating fruits already eaten), sustained attention, hand–eye coordination.	
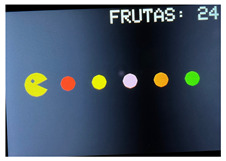
Space Invaders	A spaceship defends a colored bar from an alien that fires every 6 s. Patients must align the ship’s column (color) to intercept the alien, enhancing reaction speed, movement strength, sustained attention, and decision-making under pressure. Alien appearances are randomized. In addition to using the sensorized handle, players can use a rotatory knob to move the ship through the columns, which promotes the rehabilitation of fine psychomotor skills in the fingers.	Motor: repetitive and fast upper-limb movements; fine motor control with the rotatory knob for shooting. Cognitive: intermediate cognitive load (recognize new alien position), reaction speed, visuomotor coordination.	
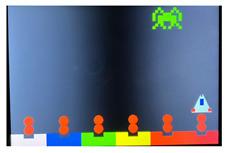

## Appendix B. Full RGB Signal Processing

This appendix presents in detail how the RGB signal from the handle’s sensor, previously introduced in Section 5.2, has been processed. The entire procedure is accompanied by examples intended to help properly understand the proposed design.

First, the six colors were calibrated, and the RGB components of each color were recorded. During the calibration process, 500 samples of each color were collected for 5 s and then averaged. The values for each color and the identifier can be seen in Table A2.

**Table A2:** Calibrated 16-bit RGB values for each color sample.

Color	Color ID	R***_c_*** (*)	G***_c_*** (*)	B***_c_*** (*)
Red	C1	5053	1076	1103
Orange	C2	7227	2326	1595
Yellow	C3	8884	4797	2659
Pink	C4	8470	3890	3029
Blue	C5	2177	2859	2356
Green	C6	2965	3028	1678
Unidentified	C0	≠	≠	≠

(*) 16-bit ADC values serve as the center off the Euclidean distance.

The following procedures were applied for signal processing. Each is intended to solve a series of problems, which are summarized below:

- Fast reading and filtering: Three quick samples are averaged to cancel sensor shot–noise and stop the color ID from the fluctuation frame-to-frame.
- Chromatic normalization: Divides by the RGB sum so that the ensuing decision depends on the hue, not on how bright the scene is.
- Euclidean distance: Compares the normalized vector to the six calibrated centers and finds the closest one in a single lightweight step.
- Threshold assignment: Ignores the “closest” class when its distance still lies outside the threshold, thereby eliminating undefined readings.
- Temporal stabilizer: Requires the new ID to persist for 120 ms before it is published, suppressing flicker caused by hand tremors or specular highlights.
- ESP-NOW transmission: Sends a stable ID every 200 ms, keeping traffic and power consumption to a minimum.

For a better understanding of signal processing, orange color readings (ID = 2) were used as an example. The different stages of processing are detailed below:

### Appendix B.1. Fast Reading and Filtering

Attenuates instantaneous noise from TCS34725: the variance decreases approximately with 1/N, preventing random color decision oscillations. N=3 raw R,G,B readings are taken 2 ms apart and each channel is averaged. This number of samples and frequency were selected to ensure real-time color changes, thereby improving the system response and not slowing down the gaming experience:

$$\bar{R}=\frac{1}{N}\sum_{k=1}^{N}R_{k},\;\bar{G}=\frac{1}{N}\sum_{k=1}^{N}G_{k},\;\bar{B}=\frac{1}{N}\sum_{k=1}^{N}B_{k}.$$

Example (orange, color ID = 2)

$$\begin{array}{cc} & {\mathrm{READ}}_{1}:\;R_{1}=7901,\;G_{1}=2667,\;B_{1}=1777\\ & {\mathrm{READ}}_{2}:\;R_{2}=7902,\;G_{2}=2866,\;B_{2}=1776\\ & {\mathrm{READ}}_{3}:\;R_{3}=7902,\;G_{3}=2865,\;B_{3}=1776 \end{array}$$

$$\begin{array}{cc} \bar{R} & =\frac{7901+7902+7902}{3}=7\;901,\\ \bar{G} & =\frac{2667+2866+2867}{3}=2\;666,\\ \bar{B} & =\frac{1777+1776+1776}{3}=1\;776. \end{array}$$

Similar sampling and timing have been found in other microcontrollers and RGB sensor applications. In [74], they averaged five consecutive RGB readings and reported that the spread between successive values decreased below three counts per channel after this consolidation. In [75], they also used the TCS34725 with 2.4 ms cycles, enabling a 100 Hz sampling rate that is noise-robust yet real-time. Collectively, these precedents show that averaging a small set of millisecond-spaced readings is a well-accepted compromise between noise attenuation and responsiveness.

### Appendix B.2. Chromatic Normalization

Chromatic normalization (conversion of raw RGB values to normalized ratios) is a well-established technique for achieving color constancy across varying illumination levels [76,77]. In this stage, the sum of the intensities ($\bar{R}+\bar{G}+\bar{B}$) is computed, and the normalized values are obtained. This step is performed because brighter or dimmer lights scale all three channels almost equally, so by dividing, those global factors are removed, and the vector $\left(r^{\prime },g^{\prime },b^{\prime }\right)$ describes only the proportion of each channel, that is, the hue. Therefore, it ensures that the classification depends on color rather than illumination level, achieving invariance to changes in brightness or shadows.

$$s=\bar{R}+\bar{G}+\bar{B},\;\left(r^{\prime },g^{\prime },b^{\prime }\right)=\left(\frac{\bar{R}}{s},\frac{\bar{G}}{s},\frac{\bar{B}}{s}\right)$$

Example (orange, color ID = 2) 

$$s=\bar{R}+\bar{G}+\bar{B}=7\;901+2\;666+1\;776=12\;667$$

$$\left(r^{\prime },g^{\prime },b^{\prime }\right)=\left(\frac{7\;901}{12\;343},\frac{2\;666}{12\;343},\frac{1\;776}{12\;343}\right)\approx \left(0.6401,\;0.2160,\;0.1439\right)$$

With the raw readings averaged, the chromatic normalization step converted $\bar{R}=7901$, $\bar{G}=2666$ and $\bar{B}=1776$ into a dimensionless triplet $\left(r^{\prime },g^{\prime },b^{\prime }\right)=\left(0.6401,0.2160,0.1439\right)$, meaning that approximately 62% of the captured light was in the red band, 23% in the green band, and 15% in the blue band. Because these three proportions always add up to one, the vector encodes only hue: a clear predominance of red, tempered by a moderate proportion of green, and very little blue, places the sample firmly in the orange region of the color space. More importantly, if the scene were more or less brightly lit, each channel would be scaled by nearly the same factor, so the proportions (and thus the perceived hue) would remain essentially unchanged. This normalization protects the classifier from changes in overall luminance and allows the subsequent distance calculation to compare the sample only to the relative RGB composition of each calibrated color center, which improves the robustness to shadows, reflections, and other luminance variations.

### Appendix B.3. Euclidean Distance

The algorithm treats color detection as a geometric problem in a three-dimensional space defined by the normalized components $\left(r^{\prime },g^{\prime },b^{\prime }\right)$. For every color class *i* (red, orange, yellow, pink, blue, and green) a reference center $\left(R_{c_{i}},G_{c_{i}},B_{c_{i}}\right)$ is stored (Table A2) and obtained during calibration with the same sensor and lighting. When a new sample is captured, firmware computes the squared Euclidean distance for each class

$$d_{i}^{2}={\left(r^{\prime }-R_{c_{i}}\;\right)}^{2}+{\left(g^{\prime }-G_{c_{i}}\right)}^{2}+{\left(b^{\prime }-B_{c_{i}}\right)}^{2}$$

and select the class that yields the smallest value. Because both the sample and the centers have already been chromatically normalized, brightness information has been removed, so the distance now reflects only the differences in hue.

This Euclidean distance metric compactly aggregates the three channel-wise deviations into a single scalar measure, facilitating lightweight, threshold-based classification in firmware without compromising real-time performance. Similar approaches have been proven to be effective in other RGB-sensor applications [78,79,80].

Example (orange, color ID = 2) 

$$\begin{array}{cc} C_{1} & \;⟶\;d^{2}=0.00802\\ C_{2} & \;⟶\;d^{2}=0.00012\\ C_{3} & \;⟶\;d^{2}=0.01567\\ C_{4} & \;⟶\;d^{2}=0.01221\\ C_{5} & \;⟶\;d^{2}=0.17918\\ C_{6} & \;⟶\;d^{2}=0.10186 \end{array}$$

The smallest value ($d^{2}=0.00012$) belongs to C2 (Color ID = 2); therefore, the classifier unambiguously assigns the sample to orange. All other distances are at least an order of magnitude larger, confirming that their chromatic proportions diverge appreciably from those of the reference. Because the decision reduces to picking the minimum of six lightweight Euclidean calculations, the procedure fits comfortably inside ESP32’s main loop without delaying the application. The geometric interpretation is equally transparent: the calibrated centers form six points in a color “cloud,” and the algorithm simply chooses the one closest to the sample. Any systematic bias of the sensor is already baked into those centers; therefore, no extra weighting factors are required to keep the six color clusters cleanly separated.

### Appendix B.4. Threshold Assignment

The Euclidean block always returns one class as the “least distant,” even when the sample is far from every center. To prevent such false positives, firmware adds a second gate. This “double-criterion” scheme (winner by minimum distance followed by threshold check) is also found in the literature, for example in [81]. They classified wood color by calculating the confidence intervals of the distances within each color cluster and setting the threshold as the upper bound. Thus, the authors rejected samples outside the confidence range of their classes.

This application of statistical thresholds is useful in embedded systems with RGB sensors; detection is only validated if the distance between the reading and the class center is below the threshold. This was noted by the authors of [82].

In this study, the second gate is defined as follows.

1. Winner search. It determines an index that minimizes the distance
  $$C^{⋆}=\arg\min_{i}d_{i}^{2},$$
  where $C^{⋆}$ denotes the optimal or the selected value. In other words, the color class whose center lies closest to the sample is in the normalized RGB space. This is the candidate color (candidateColor in the code).
2. Confidence checks. The candidate is accepted only if its distance is smaller than the empirical radius of its own class.
  $$d_{C_{i}^{⋆}}^{2}<\tau_{C_{i}^{⋆}},$$
  where $\tau_{C_{i}^{⋆}}$ is the empirical dispersion radius of class *i*. This is calculated as:
  $$\tau_{C_{i}^{⋆}}=\mu_{d^{2}}^{\left(i\right)}+k\;\sigma_{d^{2}}^{\left(i\right)},\;k=3.$$
  where:
  $$\mu_{d^{2}}^{\left(i\right)}=\frac{1}{n}\sum_{k=1}^{n}d_{ik}^{2},\;\sigma_{d^{2}}^{\left(i\right)}=\sqrt{\frac{1}{n-1}\sum_{k=1}^{n}{\left(d_{ik}^{2}-\mu_{d^{2}}^{\left(i\right)}\right)}^{2}}$$

Here, $\mu_{d^{2}}^{\left(i\right)}$ and $\sigma_{d^{2}}^{\left(i\right)}$ are the mean and standard deviation of the *n* distance samples collected for class *i* (n = 200 in this case), respectively. A separate threshold routine script runs 200 iterations (each computing the squared distance from the 3-read averaged RGB to the stored class center) to produce these n=200 samples. Choosing k=3 applies the classic three-sigma rule: for a Gaussian variable *x* with mean μ and standard deviation σ,

$$P\;\;\;\left(|x-\mu |\leq 3\sigma \right)\approx 0.9973,$$

Therefore, μ±3σ encloses 99.7 % of the in-class readings. This three-sigma threshold was also found in the literature, for example in [83,84]. Although the exact distribution of $d^{2}$ is not perfectly normal, the measured histograms are bell-shaped and sufficiently symmetric that the three-sigma bound maintains a false rejection rate below 0.3% while preserving a gap of one to two orders of magnitude to the nearest foreign class. It is trivial to compute, has a clear statistical meaning, and requires no per-color fine-tuning. Our final thresholds, $\tau_{i}=\mu_{d^{2}}^{\left(i\right)}+3\sigma_{d^{2}}^{\left(i\right)}$, map exactly to the array $\tau_{i}$[0.000252, 0.000330, 0.000128, 0.000960, 0.000848, 0.000069] hard-coded in the firmware (Table A3).

**Table A3:** Statistical derivation of class thresholds in normalized RGB space.

Color	$\mu_{d^{2}}$	$\sigma_{d^{2}}$	$\tau_{i}$ (*)
Red	2.38 × 10^−4^	5.20 × 10^−6^	2.52 × 10^−4^
Orange	3.20 × 10^−4^	3.30 × 10^−6^	3.30 × 10^−4^
Yellow	1.20 × 10^−4^	2.60 × 10^−6^	1.28 × 10^−4^
Pink	9.39 × 10^−4^	7.00 × 10^−6^	9.60 × 10^−4^
Blue	8.34 × 10^−4^	5.00 × 10^−6^	8.48 × 10^−4^
Green	0.669 × 10^−4^	1.10 × 10^−6^	0.69 × 10^−4^

(*) $\tau_{i}=\mu_{d^{2}}+3\times \sigma_{d^{2}}$.

Demanding $d_{C^{⋆}}^{2}<\tau_{C^{⋆}}$ therefore verifies that the sample falls inside the empirical confidence sphere of the chosen class and eliminates out-of-gamut or glare-induced readings that would otherwise pass as “least bad.” The extra comparison costs one if per loop but yields a classifier that is both fast and resilient.

Example(orange,colorID=2)

$$\begin{array}{cccc} d_{1}^{2} & =80.2\times 10^{-4} & >\; & \tau_{1}=2.52\times 10^{-4}\\ d_{2}^{2} & =1.20\times 10^{-4} & <\; & \tau_{2}=3.30\times 10^{-4}\;⟹\;\mathrm{accepted}\;\left(\mathrm{orange}\right)\\ d_{3}^{2} & =156.7\times 10^{-4} & >\; & \tau_{3}=1.28\times 10^{-4}\\ d_{4}^{2} & =122.1\times 10^{-4} & >\; & \tau_{4}=9.60\times 10^{-4}\\ d_{5}^{2} & =1791.8\times 10^{-4} & >\; & \tau_{5}=8.48\times 10^{-4}\\ d_{6}^{2} & =1018.6\times 10^{-4} & >\; & \tau_{6}=0.69\times 10^{-4} \end{array}$$

The orange class not only wins the argmin race but also lies well inside its tolerance circle, whereas the remaining colors all exceed their respective limits by at least one order of magnitude. This dual criterion eliminates ambiguous or out-of-gamut readings, reduces false positives, and keeps the classifier reliable even under sensor noise or glare.

### Appendix B.5. Temporal Stabilizer

The last stage prevents the output from flickering when small hand tremors cause the sensor to oscillate between two neighboring classes. The following two state variables are involved.

$$C^{⋆}\left(t\right):\;\mathrm{validated}\;\mathrm{candidate}\;\mathrm{at}\;\mathrm{time}\;t,$$

$$\hat{C}\left(t\right):\;\mathrm{stable}\;\mathrm{color}\;\mathrm{ID}\;\mathrm{currently}\;\mathrm{published}.$$

In firmware, these are the variables candidateColor and lastColorStable. The stabilizer applies a dwell–time hysteresis:

1. Match. If the freshly validated candidate $C^{⋆}\left(t\right)$ coincides with the official color $\hat{C}\left(t\right)$ the reading simply confirms that the current state and internal timer are reset.
2. Mismatch. If $C^{⋆}\left(t\right)\neq \hat{C}\left(t\right)$, the candidate is stored and a timer starts. Only if the same $C^{⋆}$ persists for
  $$T_{\mathrm{stab}}=120\;\;\mathrm{ms}$$
  (approximately six consecutive main–loop iterations of $2.000\times 10^{1}$ ms each) is promoted to the new official value,
  $$\hat{C}\left(t+T_{\mathrm{stab}}\right)=C^{⋆}\left(t\right).$$
  Otherwise, the system maintains the previous $\hat{C}\left(t\right)$.

The chosen window of 120 ms is sufficiently long to outlive single-frame glitches caused by micro-vibrations, surface reflections, or momentary shadows. This type of temporal stabilizer is also found in the literature [85,86].

### Appendix B.6. ESP-NOW Transmission

Every 200 ms, the structure {color_id, game_mode} was sent. The game_mode indicates whether the sensorized handle is in use, so that if the main menu is active, no unnecessary readings or transmissions occur, avoiding extra energy consumption.

### Appendix B.7. Synthesis

These procedures make reading the current color as robust as possible. They solve problems caused by erroneous colors or adverse conditions. The following example uses a black cardboard to demonstrate the significant changes in the signal, and a returned ID of 0.

**Example: black cardboard (ID = 0)**

1. Fast reading and filtering
  $$\begin{array}{cc} & {\mathrm{READ}}_{1}:R_{1}=396,\;G_{1}=421,\;B_{1}=313\\ & {\mathrm{READ}}_{2}:R_{2}=396,\;G_{2}=420,\;B_{2}=312\\ & {\mathrm{READ}}_{3}:R_{3}=396,\;G_{3}=420,\;B_{3}=312 \end{array}$$
  $$\bar{R}=396,\;\bar{G}=420,\;\bar{B}=312.$$
2. Chromatic normalization
  $$s=\bar{R}+\bar{G}+\bar{B}=396+420+312=1\;128$$
  $$\left(r^{\prime },g^{\prime },b^{\prime }\right)=\left(\frac{396}{1\;128},\frac{420}{1\;128},\frac{312}{1\;128}\right)\approx \left(0.3511,\;0.3723,\;0.2766\right).$$
3. Euclidean distance & threshold test. For each class *i* the firmware computes the squared distance $d_{i}^{2}$ and immediately checks it against the class threshold $\tau_{i}$:
  $$d_{i}^{2}={\left(r^{\prime }-R_{c_{i}}\right)}^{2}+{\left(g^{\prime }-G_{c_{i}}\right)}^{2}+{\left(b^{\prime }-B_{c_{i}}\right)}^{2}\;\mathrm{accept}\;\mathrm{if}\;d_{i}^{2}<\tau_{i}.$$
  The smallest distance was $d_{6}^{2}=0.00511$, but it still exceeded its threshold by two orders of magnitude $\left(d_{6}^{2}>\tau_{6}\right)$. Therefore, the candidate is rejected, and the classifier outputs ID=0 (“undefined color”).

**Table A4:** Example for a color ID = 0.

Class	$d_{i}^{2}$	$\tau_{i}$
$C_{1}\;\left(\mathrm{red}\right)$	1862.2 $\times \;10^{-4}$	2.52 $\times \;10^{-4}$
$C_{2}\;\left(\mathrm{orange}\right)$	1329.6 $\times \;10^{-4}$	3.30 $\times \;10^{-4}$
$C_{3}\;\left(\mathrm{yellow}\right)$	562.8 $\times \;10^{-4}$	1.28 $\times \;10^{-4}$
$C_{4}\;\left(\mathrm{pink}\right)$	603.9 $\times \;10^{-4}$	9.60 $\times \;10^{-4}$
$C_{5}\;\left(\mathrm{blue}\right)$	51.8 $\times \;10^{-4}$	8.48 $\times \;10^{-4}$
$C_{6}\;\left(\mathrm{green}\right)$	51.1 $\times \;10^{-4}$	0.69 $\times \;10^{-4}$

This example illustrates how the cascade of filtering, normalization, distance computation and thresholding prevents a dark background (even one that momentarily resembles green in chromatic terms) from being mistaken for any of six calibrated classes.
